# The evolution of survival of pulmonary arterial hypertension over 15 years

**DOI:** 10.1002/pul2.12137

**Published:** 2022-10-01

**Authors:** Paul M. Hendriks, Diederik P. Staal, Liza D. van de Groep, Leon M. van den Toorn, Prewesh P. Chandoesing, Robert M. Kauling, Hans‐Jurgen Mager, Annemien E. van den Bosch, Marco C. Post, Karin A. Boomars

**Affiliations:** ^1^ Department of Respiratory Medicine Erasmus University Medical Center Rotterdam The Netherlands; ^2^ Department of Cardiology Erasmus University Medical Center Rotterdam The Netherlands; ^3^ Department of Cardiology St. Antonius Hospital Nieuwegein The Netherlands; ^4^ Department of Respiratory Medicine St. Antonius Hospital Nieuwegein The Netherlands; ^5^ Department of Cardiology University Medical Center Utrecht Utrecht The Netherlands

**Keywords:** prognosis, pulmonary arterial hypertension, pulmonary hypertension, survival

## Abstract

The prognosis of pulmonary arterial hypertension (PAH) remains dismal. Over the years, multiple therapeutic advances have been introduced. This study evaluates the evolution of PAH survival over the past 15 years. We included 293 consecutive adult patients diagnosed with PAH between 2005 and 2019 (median age: 61.8 years, 70.3% female). Patients were divided into three cohorts based on the time of diagnosis: 2005–2009, 2010–2014, and 2015–2019 (2005–2009: *n* = 56; 2010–2014: *n* = 111; 2015–2019: *n* = 126). Transplant‐free survival was measured from the date of right heart catheterization until patients reached the composite endpoint of lung transplant or death. Multivariable cox‐pulmonary hypertension regression was used to study the effect of the time of diagnosis. The final cox model was fitted in both younger and older patients to evaluate the difference between these groups. During a median follow‐up time of 4.1 (interquartile range: 2.2–7.3) years, 9 patients underwent lung transplantation and 151 patients died. The median overall transplant‐free survival was 6.2 (5.5–8.0) years. Patients older than 56 years at baseline who were diagnosed in 2005–2009 showed better survival compared to patients diagnosed in 2010–2014 and 2015–2019 with an adjusted hazard ratio of, respectively, 2.12 (1.11–4.03) and 2.83 (1.41–5.69). Patients younger than 56 years showed neither an improved nor deteriorated survival over time. In conclusion, survival in patients with PAH did not improve over time, despite more available therapeutic options. This might be partly due to the changed demographic characteristics of the PAH patients and a still important diagnostic delay.

## INTRODUCTION

Pulmonary arterial hypertension (PAH) is a progressive disease of the pulmonary vasculature. It affects approximately 7 per million people per year.^1^, ^2^ The diagnosis of PAH is confirmed by right heart catheterization and is characterized by a mean pulmonary artery pressure (mPAP) of ≥25 mmHg, a pulmonary capillary wedge pressure (PCWP) ≤ 15 mmHg, and pulmonary vascular resistance (PVR) ≥ 3 WU according to the 2015 European Society of Cardiology/European Respiratory Society (ESC/ERS) guidelines.^3^ Underlying pathophysiological mechanisms include pulmonary arteriolar remodeling leading to an increased PVR. This will ultimately result in right ventricular failure and death. PAH can be divided into further subgroups, based on the underlying cause or disease (e.g., genetic mutations, connective tissue disease, and congenital heart disease). If no underlying cause can be determined, it is classified as idiopathic pulmonary arterial hypertension (IPAH).^3^

Multiple registries have been established over the past decades. The first one to report on survival was published in 1991 and reported a 5‐year survival of 34% in incident patients with IPAH. More recent reports showed improved survival rates. Humbert et al.^4^ studied patients with familial, idiopathic, or anorexigen‐associated PAH and found a 3‐year survival of 67%. Benza et al.^5^ and Zhang et al.^6^ found a 3‐year survival of, respectively, 75.1% and 68%. Despite this improvement, the prognosis remains dismal. Over the last 2 decades, new PAH‐specific medication has become available, affecting different pathophysiological pathways and treatment guidelines have been adapted accordingly.^3^

Not only did the therapeutic options change, the epidemiological and demographic characteristics of the PAH patient changed over time too. Registries observed that the age of the patients diagnosed increased over time and that survival of elderly patients is worse compared to younger patients.^7^ Furthermore, due to improved awareness among physicians and screening programs for PAH for specific groups, for example, patients suffering from systemic sclerosis, more patients have been diagnosed.

Limited data exist on how the prognosis of PAH changed by this evolving landscape. The primary objectives of this study were 1) to evaluate the evolution of transplant‐free survival in patients with PAH over the past 15 years; 2) to provide an up‐to‐date overview of mortality; and 3) to evaluate the (development of) treatment strategies in patients with PAH.

## METHODS

### Study population

In this study, all consecutive incident adult patients diagnosed with PAH between 2004 and 2019 from two pulmonary hypertension (PH) expertise centers in the Netherlands were screened for inclusion. All patients were treatment naïve at baseline and were treated in accordance with the ESC/ERS guidelines for pulmonary hypertension after the diagnosis PAH was established.^3^

All patients underwent an inpatient screening visit, during which the following tests were performed: 6‐min walking test (6MWD), 12‐lead electrocardiogram, transthoracic echocardiography, venous blood sampling, pulmonary function tests, lung perfusion scintigraphy, chest computed tomography scan, and a right heart catheterization with a fluid challenge when diastolic dysfunction was suspected. The date of right heart catheterization was considered the date of diagnosis. PAH was defined as a mPAP of ≥25 mmHg and PVR ≥ 3 WU with a PCWP of ≤15 mmHg, according to the contemporaneous guidelines.^3^ The diagnosis of PAH was made by a multidisciplinary team, including specialized pulmonary physicians, cardiologists, and specialized PH‐nurses. Patients who did not classify as PH World Health Organization (WHO) Class 1 either by hemodynamic parameters or by the opinion of the multidisciplinary team were excluded.

The primary endpoint was a composite endpoint of all‐cause mortality or lung transplantation. The number of patients on PH‐specific mono‐, dual‐, or triple therapy was used as a secondary endpoint. The endpoints were evaluated at 1, 3, 5, and 10 years after diagnosis. To compare the evolution of the primary and secondary endpoints over time, patients were divided into three cohorts based on their date of diagnosis (2005–2009, 2010–2014, and 2015–2019).

### Data collection

Baseline demographic data, type of PAH, 6MWD, N‐terminal pro‐brain natriuretic peptide (NT‐proBNP) level, comorbidities (arterial hypertension, atrial fibrillation, chronic obstructive pulmonary disease [COPD], coronary artery disease, and diabetes mellitus), and hemodynamic measurements were retrieved for this study. We calculated the French registry noninvasive risk score based on the number of low‐risk criteria (New York Heart Association [NYHA]‐class I or II, 6MWD > 440 m, and NT‐pro‐BNP < 300 pg/ml).^8^ The type and number of PAH‐specific therapy were evaluated at 1, 3, and 5 years after inclusion. Survival of all patients was checked using the Municipal Population Register. Data on survival status was 100% complete. The online electronic case report form PAHTool was used to collect and store data (PAHTool version 4.3.5947.29411; Inovoltus).

### Statistical analysis

Continuous variables were presented as mean ± standard deviation or median (interquartile range) depending on their distribution. Categorical variables were presented as counts (percentage). Continuous variables were compared using an unpaired *t*‐test or Mann–Whitney *U* test depending on their distribution and one‐way analysis of variance when more than two subgroups were compared. Categorical data were compared using a *χ* ^2^ test or Fisher's exact test.

Transplant‐free survival was measured from the date of right heart catheterization until patients reach the composite endpoint. Survival curves were obtained using the Kaplan–Meier estimator stratified according to time cohort. Patients who did not complete the 3‐ or 5‐year follow‐up in the cohort 2015–2019 were right‐censored on the first of July 2021. Transplant‐free survival between time cohorts was compared using a logrank test. A multivariable cox proportional hazards model was used to analyze the association between the time of diagnosis and transplant‐free survival when adjusted for other clinical characteristics. Variables were selected using stepwise selection with a significance level of *p* < 0.2. Missing data were imputed using multiple imputations via chained equations and 20 imputed datasets were generated. Predictive mean matching was used for numeric variables and logistic regression for binary variables. Supporting Information: Table S1 provides an overview of missing data per predictor. Variables with more than 15% missing data were not imputed. Given the trend in the literature that the epidemiology of PAH seems to be changing and that the median age is increasing, a sensitivity analysis was performed to determine the optimal cutoff point for age and the final cox‐model was fitted additionally to both the younger and the older patients. To avoid overfitting the cox‐PH models in the age‐based subpopulations, further forward selection was performed to construct a model with the appropriate number of variables. The proportional hazards assumption was evaluated using Schoenfeld residuals.

Statistical analysis was performed using SPSS (IBM Corp., released 2017, IBM SPSS Statistics for Windows, version 25.0. IBM Corp.) and R (R Core Team (2017). R: A language and environment for statistical computing. R Foundation for Statistical Computing. URL https://www.R-project.org/) using the packages “survival,” “ggplot2,” and “mice.”

## RESULTS

### Baseline characteristics

A total of 293 patients with PAH were included in this study (cohorts 2005–2009: *n* = 56; 2010–2014: *n* = 111; and 2015–2019: *n* = 126). There was no significant difference in the amount of included patients between centers (Supporting Information: Table S2). The median age over all cohorts was 61.8 years and 70.3% were female (Table 1). The proportion of patients diagnosed with IPAH was significantly higher in the cohort of patients diagnosed between 2005 and 2009 (2005–2009: 46.4%; 2010–2014: 20.7%; and 2015–2019: 31.7%; *p* = 0.003). Significantly less patients with PAH due to connective tissue disease were present in the cohort diagnosed between 2005 and 2009 (2005–2009: 16.1%; 2010–2014: 40.5%; and 2015–2019: 40.5%; *p* = 0.003). Furthermore, 15.4% of the patients were diagnosed with PAH due to congenital heart disease, 6.5% with portopulmonary hypertension, 4.4% with heritable PAH, 3.8% with the pulmonary venous occlusive disease, 3.1% with drugs and toxins induced, and 0.7% secondary to HIV infection. No significant differences were found between subgroups regarding hemodynamic parameters. The median mPAP was 46 (37–56) mmHg, with a median PCWP of 9 (6–13) mmHg and PVR of 7.4 (4.9–10.8) WU. No significant differences were found between cohorts in comorbidities (arterial hypertension, atrial fibrillation, COPD, coronary artery disease, and diabetes mellitus).

**Table 1:** baseline characteristics of the whole cohort and each time cohort

	All cases (*n* = 293)	2005–2009 (*n* = 56)	2010–2014 (*n* = 111)	2015–2019 (*n* = 126)	*p* Value
*Demographic characteristics*					
Female sex (%)	206 (70.3)	43 (76.8)	75 (67.6)	88 (69.8)	0.463
Age, years	61.8 (46.3–70.6)	57.2 (47.3–69.5)	61.9 (43.2–70.7)	63.1 (49.0–71.5)	0.578
BMI, kg/m^2^	27.1 ± 5.8	27.1 ± 5.9	26.4 ± 5.3	27.6 ± 6.1	0.221
*Disease subtype*					
Idiopathic (%)	89 (30.4)	26 (46.4)	23 (20.7)	40 (31.7)	0.003
Heritable (%)	13 (4.4)	3 (5.4)	4 (3.6)	6 (4.8)	0.850
Drugs and toxins induced (%)	9 (3.1)	0 (‐)	7 (6.3)	2 (1.6)	0.037
Connective tissue disease (%)	105 (35.8)	9 (16.1)	45 (40.5)	51 (40.5)	0.003
Systemic lupus erythematosus (%)	9 (8.7)	1 (11.1)	3 (6.8)	5 (9.8)	
Systemic sclerosis (%)	72 (69.2)	5 (55.6)	33 (75.0)	34 (66.7)	
Sjögren syndrome (%)	5 (4.8)	1 (11.1)	1 (2.3)	4 (7.8)	
Mixed connective tissue disease (%)	9 (8.7)	1 (11.1)	2 (4.5)	6 (11.8)	
Other (%)	9 (8.7)	1 (11.1)	5 (11.4)	2 (3.9)	
HIV infection (%)	2 (0.7)	1 (1.8)	1 (0.9)	0 (–)	0.377
Portopulmonary hypertension (%)	19 (6.5)	3 (5.4)	7 (6.3)	9 (7.1)	0.899
Congenital heart disease (%)	45 (15.4)	13 (23.2)	19 (17.1)	13 (10.3)	0.068
PVOD and/or pulmonary capillary hemangiomatosis (%)	11 (3.8)	1 (1.8)	5 (4.5)	5 (4.0)	0.674
*Clinical characteristics*					
Comorbidities					
Arterial hypertension (%)	122 (41.6)	17 (30.4)	44 (39.6)	61 (48.4)	0.057
Atrial fibrillation (%)	42 (14.3)	9 (16.1)	19 (17.1)	14 (11.1)	0.400
COPD (%)	48 (16.4)	10 (17.9)	22 (19.8)	16 (12.7)	0.331
Coronary artery disease (%)	34 (11.6)	4 (7.1)	11 (9.9)	19 (15.1)	0.227
Diabetes mellitus (%)	62 (21.2)	11 (19.6)	21 (18.9)	30 (23.8)	0.603
NYHA class					0.159
‐ I (%)	13 (4.4)	2 (3.6)	4 (3.6)	7 (5.6)	
‐ II (%)	88 (30.0)	18 (32.1)	40 (36.0)	30 (23.8)	
‐ III (%)	158 (53.9)	34 (60.7)	52 (46.8)	72 (57.1)	
‐ IV (%)	34 (11.6)	2 (3.6)	15 (13.5)	17 (13.5)	
NT‐proBNP, pg/ml	1077 (315–2670)	1089 (234–3029)	1196 (392–2799)	975 (288–2424)	0.719
6MWD, m	339 ± 130	355 ± 136	345 ± 130	327 ± 127	0.423
French registry noninvasive risk score^a^					0.816
‐ 0 low‐risk criteria (%)	91 (43.8)	16 (43.2)	33 (42.4)	42 (45.2)	
‐ 1 low‐risk criteria (%)	71 (34.1)	10 (27.1)	27 (34.6)	34 (36.6)	
‐ 2 low‐risk criteria (%)	35 (16.8)	9 (24.3)	14 (17.9)	12 (12.8)	
‐ 3 low‐risk criteria (%)	11 (5.3)	2 (5.4)	4 (5.1)	5 (5.4)	
*Hemodynamic data*					
mRAP, mmHg	9 (6–13)	10 (7–16)	8 (6–12)	8 (5–12)	0.274
sPAP, mmHg	73 (60–90)	82 (63–100)	73 (61–87)	71 (57–89)	0.056
dPAP, mmHg	30 (23–38)	30 (22–40)	30 (24–40)	27 (23–35)	0.246
mPAP, mmHg	46 (37–56)	50 (38–62)	47 (39–56)	45 (36–52)	0.064
PCWP, mmHg	9 (6–13)	11 (8–13)	11 (8–12)	10 (7–12)	0.162
CO, L/min	4.9 ± 1.7	4.9 ± 1.8	4.9 ± 1.8	5.0 ± 1.6	0.934
PVR, WU	7.4 (4.9–10.8)	8.7 (5.2–13.6)	7.9 (4.3–11.2)	6.8 (5.0–9.8)	0.271

^a^ Low‐risk criteria: NYHA‐class I or II, 6MWD > 440 m, and NT‐pro‐BNP <300 pg/ml or <35.7 pmol/L.

Abbreviations: 6MWD, 6 min walking distance; BMI, body mass index; CO, cardiac output; COPD, chronic obstructive pulmonary disease; dPAP, diastolic pulmonary arterial pressure; mPAP, mean pulmonary arterial pressure; mRAP, mean right atrial pressure; NT‐proBNP, N‐terminal pro‐brain natriuretic peptide; NYHA, New York Heart Association; PCWP, pulmonary capillary wedge pressure; PVOD, pulmonary veno‐occlusive disease; PVR, pulmonary vascular resistance; sPAP, systolic pulmonary arterial pressure.

### Therapy strategies

**Figure 1:**
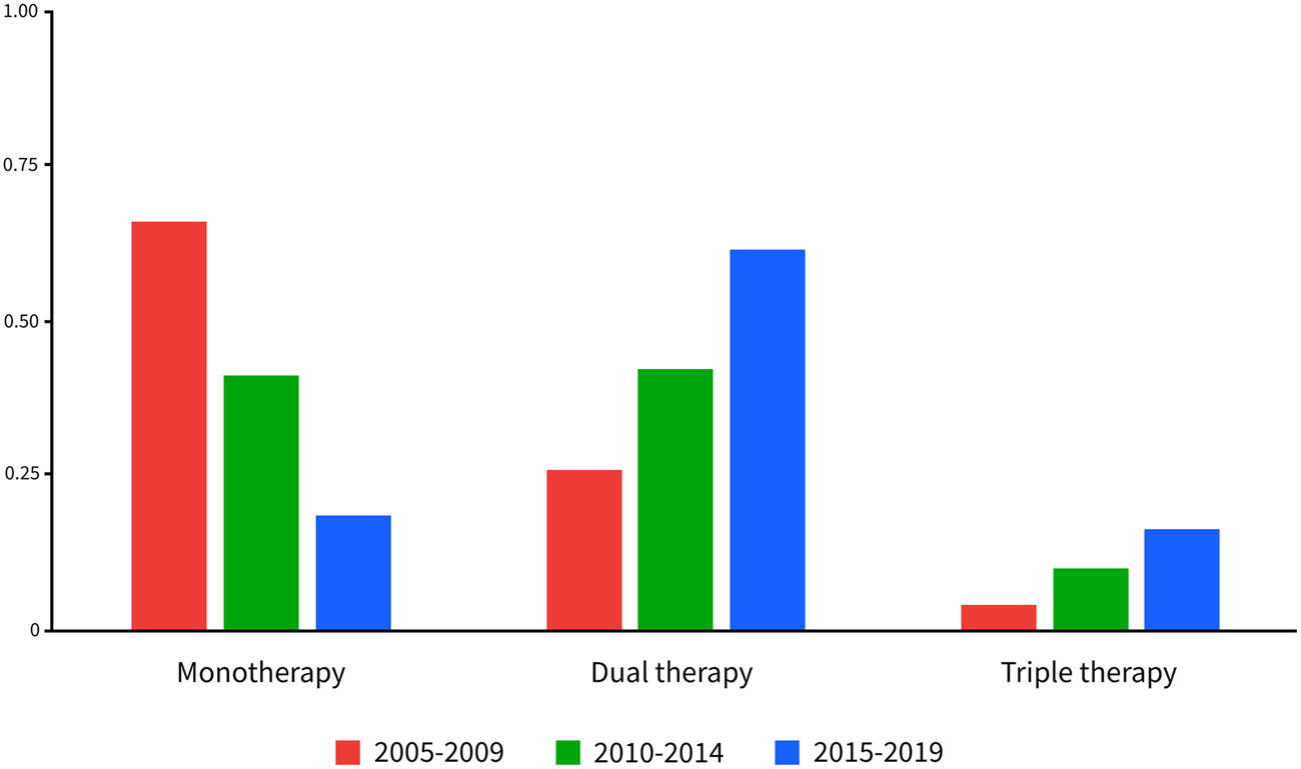
Number of pulmonary arterial hypertension‐specific drugs per time cohort.

**Figure 2:**
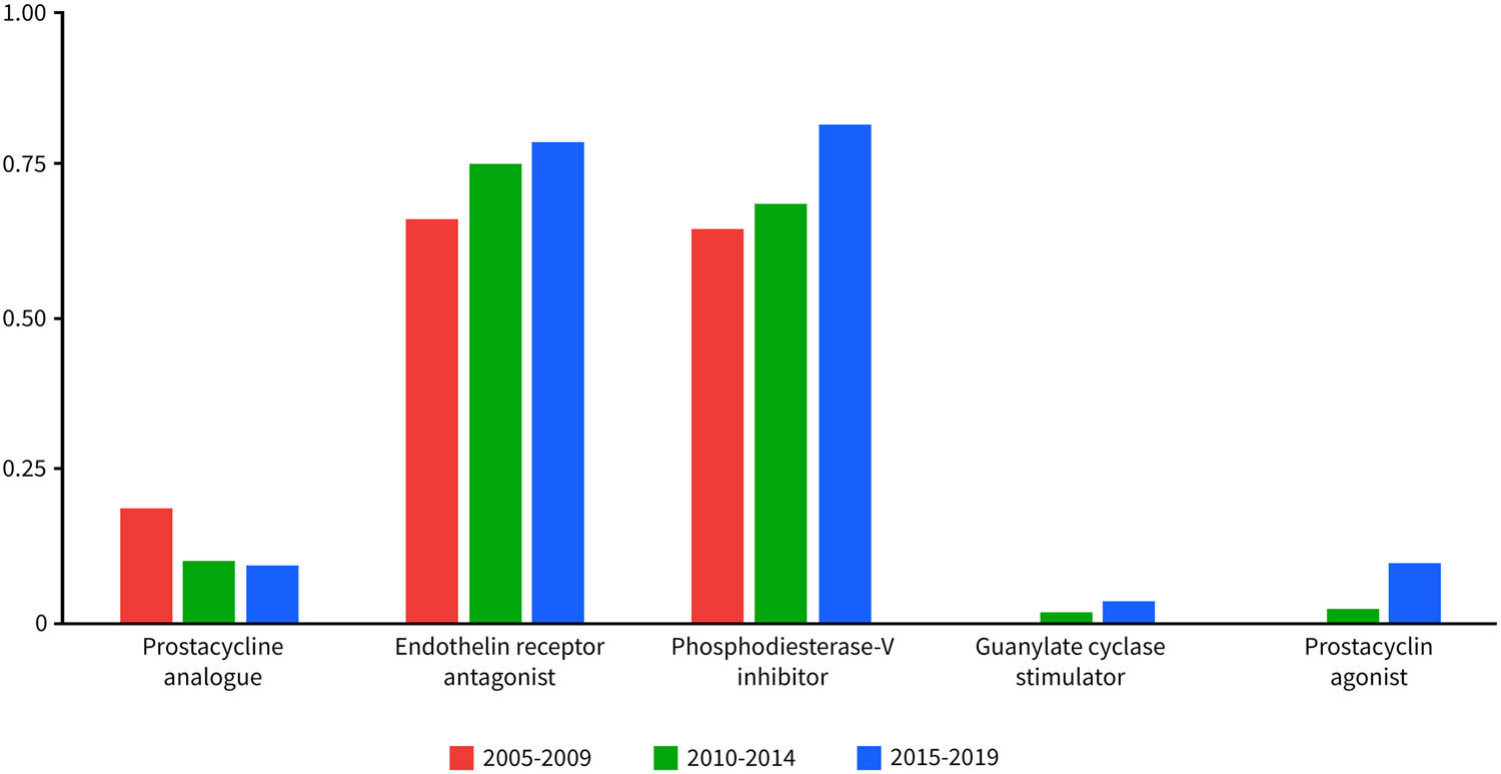
Type of pulmonary arterial hypertension‐specific medications are used per time cohort.

Figure 1 shows the proportion of patients treated with mono, dual, or triple PAH‐specific therapy 1 year after diagnosis. The number of patients treated with monotherapy decreased significantly over time (2005–2009: 66.0%; 2010–2014: 41.1%; and 2015–2019: 18.3%). The number of patients on dual or triple combination therapy with PAH‐specific medication increased over time. The number of patients on dual therapy 1 year after diagnosis increased from 26.0% in patients diagnosed between 2005 and 2009 to 61.5% in patients diagnosed between 2015 and 2019. Figure 2 illustrates the proportion of the use of PAH‐specific medication 1 year after diagnosis over time. The use of endothelin‐1 receptor antagonists and phosphodiesterase‐V inhibitors increased over time. The proportion of patients who were treated with prostacyclin analogues, intravenously or subcutaneously decreased over time almost by half (2005–2009: 18.4% vs. 2015–‐2019: 9.6%). New treatment options like guanylate cyclase stimulators and prostacyclin receptor agonists increased to, respectively, 3.6% and 9.6% in patients diagnosed between 2015 and 2019.

### Survival

**Figure 3:**
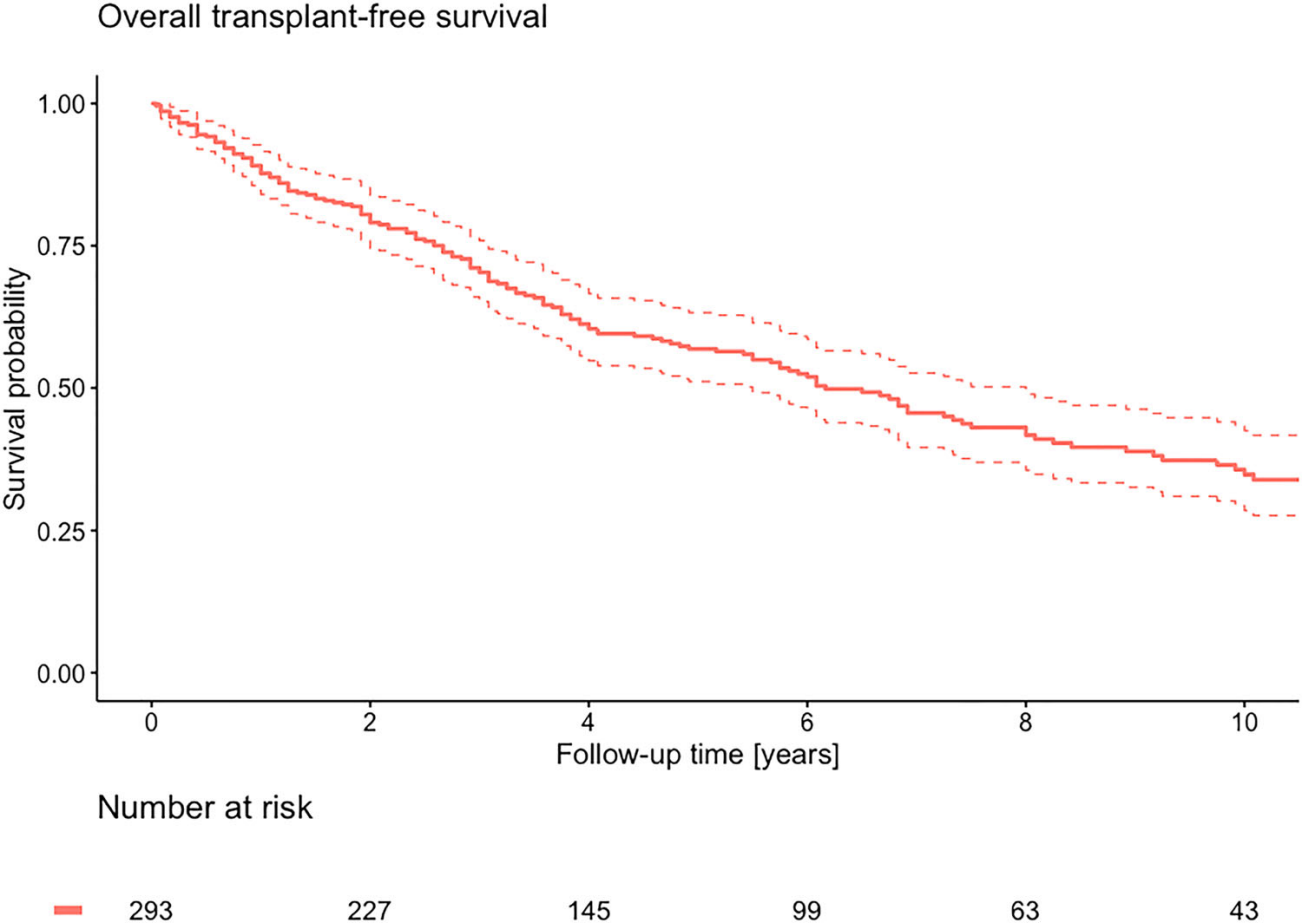
Kaplan–Meier curve of overall survival.

**Figure 4:**
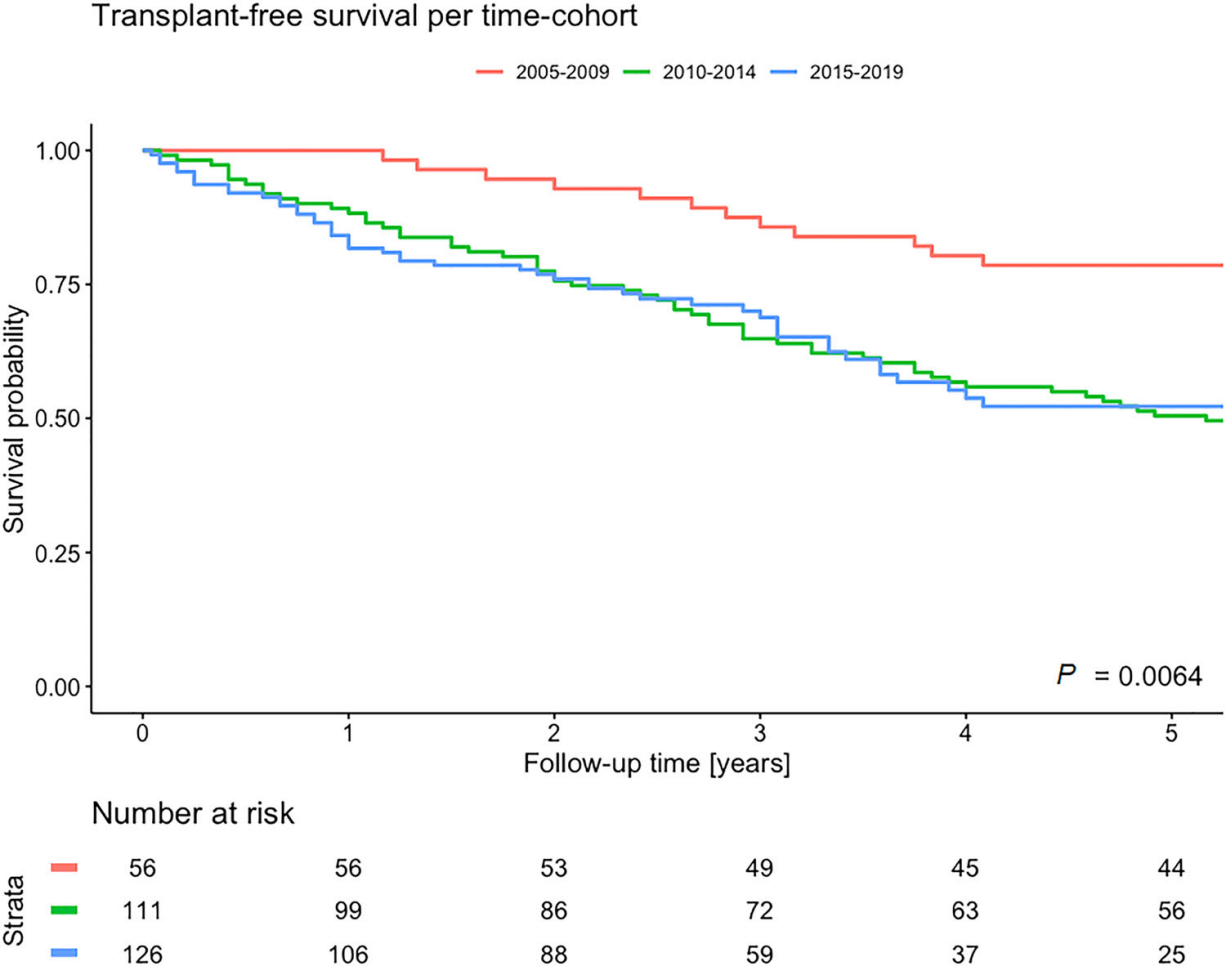
Kaplan–Meier curve of survival stratified per time cohort.

During a median follow‐up time of 4.1 (2.2–7.3) years, 9 patients underwent lung transplantation and 151 patients died. A detailed overview of the causes of death per time and age cohort can be found in Supporting Information: Table S3. The median overall transplant‐free survival was 6.2 (5.5–8.0) years with a 1‐, 3‐, and 5‐year survival rate of, respectively, 88%, 71%, and 57% (Figure 3). The 10‐year transplant‐free survival rate was 35%. Transplant‐free survival was significantly better among patients diagnosed in 2005–2009 with a 5‐year transplant‐free survival rate of 78% compared to 51% in patients diagnosed between 2010 and 2014 and 52% between 2015 and 2019 (Figure 4). No significant difference in transplant‐free survival rate was found between patients diagnosed in 2010–2014 and 2015–2019 (*p* = 0.964).

**Figure 5:**
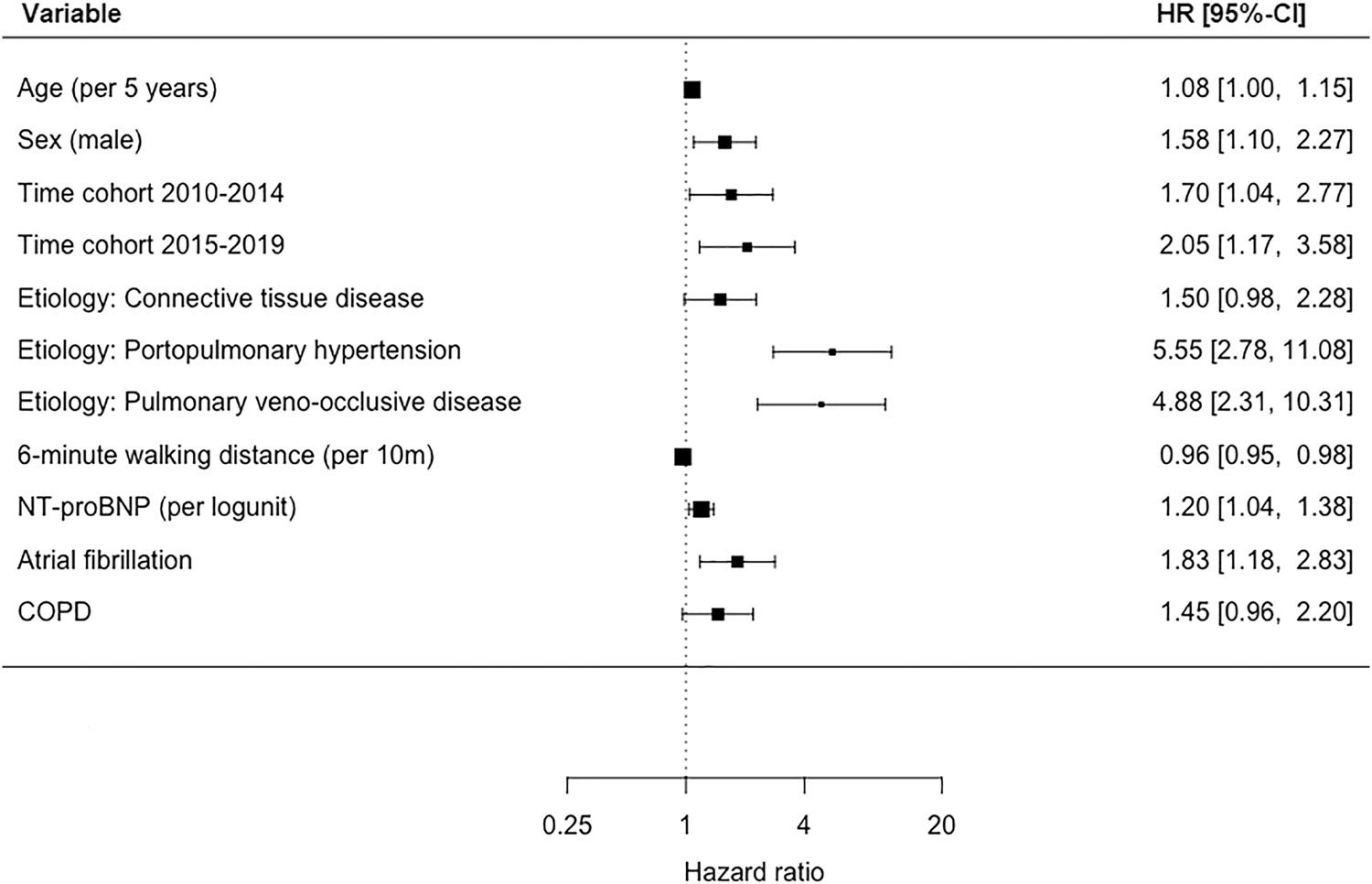
Forest plot of multivariable cox‐PH regression of total population. COPD, chronic obstructive pulmonary disease; NT‐proBNP, N‐terminal pro‐brain natriuretic peptide

**Figure 6:**
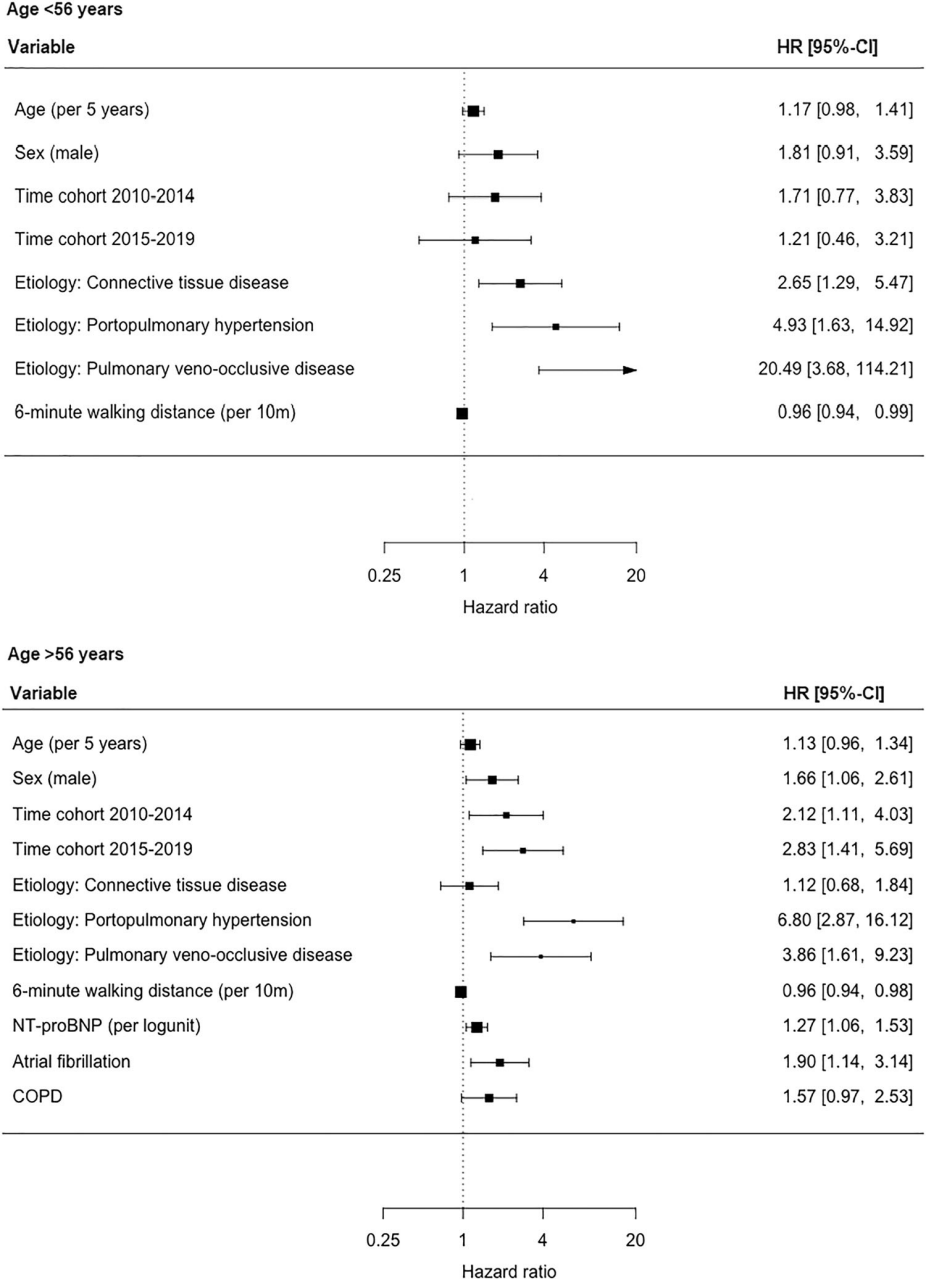
Forest plots of multivariable cox‐PH regression of subpopulations based on age. CI, confidence interval; COPD, chronic obstructive pulmonary disease; HR, hazard ratio; NT‐proBNP, N‐terminal pro‐brain natriuretic peptide.

The univariable cox‐PH regression models can be found in Supporting Information: Figure S1. Overall, patients diagnosed between 2010–2014 and 2015–2019 had a significantly worse transplant‐free survival compared with patients diagnosed between 2005 and 2009 with a hazard ratio (HR) of, respectively, 1.70 (interquartile range [IQR]: 1.04–2.77) and 2.05 (IQR: 1.17–3.58) when adjusted for age, sex, etiology, 6MWD, and NT‐proBNP (Figure 5). The optimal cutoff point for age was 56 years. Transplant‐free survival did not differ between time cohorts in patients younger than 56 years (Figure 6). In patients over 56 years, like in the overall population, transplant‐free survival was significantly worse in patients diagnosed between 2010–2014 and 2015–2019 with adjusted HRs of, respectively, 2.12 (1.11–4.03) and 2.70 (1.41–5.69).

## DISCUSSION

This study demonstrated a median overall transplant‐free survival of 6.2 years with a 5‐year transplant‐free survival rate of 57% in incident patients with PAH. We observed a 10‐year transplant‐free survival of 35%. Treatment strategies changed over time, with an increase in the number of patients on combination therapy with PAH‐specific medication. Despite major developments in the available PAH medication and treatment strategies, we did not observe an improvement in transplant‐free survival over time.

The overall transplant‐free survival improved compared to the U.S. NIH registry.^9^ The 1‐, 3‐, and 5‐year transplant‐free survival rates in our study show a slight improvement when compared with the survival of patients with PAH in earlier registries.^4^, ^5^, ^10^, ^11^, ^12^, ^13^ Recently, the investigators of pulmonary hypertension associated registry (PHAR) reported 1‐ and 3‐year survival of 90.1% and 79.4%, compared to which survival in our cohort was slight worse.^14^ However, the PAH patients included in our study were 6 years older on average compared to the patients included in the PHAR. Furthermore, the PAH patients in our study were consecutive incident, treatment‐naïve patients while in the PHAR both incident and prevalent patients were included and only 14% of the patients were treatment naïve. The time from diagnosis to study enrollment in prevalent patients could introduce survivor bias and overestimate survival in prevalent patients. In this study, we only included incident patients, which provides a better representation of the newly diagnosed patient. The French registry performed a subgroup analysis on incident patients with a 3‐year survival rate of 55% compared to which the survival observed in this study is an improvement.^4^ In our cohort, a rather large proportion is in NYHA‐Class III or IV at the time of diagnosis, which is consistent with the literature. This does, however, highlight that patients are still being diagnosed in advanced disease stages with severe symptoms. This could attribute to the, unfortunately, still rather poor prognosis and lack of improvement.

Comparing our three‐time cohorts, we did not observe an improvement in transplant‐free survival over time. Transplant‐free survival rates were significantly worse in patients diagnosed in 2010–2019 when compared with patients diagnosed in 2005–2009 when adjusted for baseline differences in etiology, comorbidities, and clinical prognostic factors. Ling et al. investigated the evolution of survival of PAH patients between 2001 and 2009. They found no difference in overall survival between the investigated time cohorts. Only when adjusted for age, functional class, 6MWD, pulmonary diffusion capacity, mean right atrial pressure, and cardiac index, a better survival was found in patients diagnosed between 2007 and 2009 compared to 2001 and 2003 (HR: 1.96; *p* = 0.019). Our study covers another and a broader range of therapeutic advances, evolution in treatment strategies, and new guideline implementations. When we compared the effect of the time cohort in patients older and younger than 56 years at the time of diagnosis, a significantly worse transplant‐free survival rate was observed in patients >56 years old. However, in our younger patients, no significant effect on transplant‐free survival over time was observed.

The number of patients in the cohorts 2010–2014 and 2015–2019 increased by more than twofold compared to the time cohort 2005–2009. There were no systematic differences in patient recruitment within or between centers. In 2009, a study conducted in the Netherlands regarding the screening of patients with systemic sclerosis for PAH was published.^15^ This study was followed by the development of the DETECT screening algorithm for PAH detection in patients with systemic sclerosis.^16^ Consequently, screening protocols for PAH, especially for patients suffering from systemic sclerosis were implemented. Combined with increased awareness about PAH among physicians, this resulted in an increase in referrals to PH expert centers. In our cohort, we observed an increase of patients diagnosed with PAH due to systemic sclerosis from 5 patients in 2005–2009 to 34 patients in 2015–2019. In addition to that, referrals of patients diagnosed with portal hypertension for screening of PH increased. Patients with PAH associated with systemic sclerosis or associated with portal hypertension have worse survival.^13^ Assuming that screening leads to an earlier diagnosis, it would be expected that survival would increase because of early treatment initiation.

We assume that the increase in the proportion of patients with systemic sclerosis is attributed to the lack of improved survival. However, even after adjusting in multivariable analysis for connective tissue disease as etiology, the prognosis remained worse in the time period 2010–2019. It is likely that survival is limited by other factors than solely PAH in these patients. The PHAROS study evaluated survival in 160 patients with systemic sclerosis and PAH or who were at risk of PAH. They found survival rates at 1 and 3 years of, respectively, 95% and 83%, which is better than survival in our study.^17^ However, this study included a significant number of patients that did not meet the hemodynamic criteria for the diagnosis of PAH.

Multiple studies described a change in the epidemiological landscape of PAH. The median age of diagnosis is increasing in patients with both PAH and IPAH.^1^, ^7^, ^18^, ^19^ Our study shows the same trend, although not significant. Shapiro et al.^19^ described a higher PCWP, diastolic pulmonary arterial pressure, transpulmonary gradient, and lower 6MWD and PVR in elderly patients over 65 years old with IPAH. Furthermore, the survival in elderly patients with IPAH and PCWP ≤15 mmHg was significantly worse than in younger patients.^7^, ^19^

Elderly patients present with more comorbidities such as ischemic heart disease, systemic hypertension, atrial fibrillation, diabetes mellitus, and hypothyroidism which could limit their prognosis compared with younger patients.^1^ Diabetes mellitus type 2 is associated with worse right ventricular function and survival.^20^, ^21^, ^22^ Hypertension, diabetes, obesity, and COPD are associated with a decreased 6MWD.^23^ Furthermore, comorbidities can complicate the evaluation of disease progression and therapeutic response.^24^

Time‐to‐diagnosis in elderly patients is longer and they are diagnosed at a more advanced stage of disease.^7^ Comorbidities can mask the diagnosis of PAH and thereby contribute to diagnostic delay.^24^ Elderly patients with PAH may also show signs of WHO‐II and WHO‐III class PH.^25^ This not only poses a diagnostic challenge but might also affect survival. Very limited evidence is available on whether such patients benefit from treatment with PAH‐specific medication. It could be reasonable that a possible increase of these “atypical” PAH patients attributed to the lack of improvement in survival.^26^ This study suggests that the transplant‐free survival of the elderly patients with PAH did not improve over time and may even have deteriorated, even after adjusting for comorbidities.

Treatment options and therapeutic strategies changed over time. The use of dual or triple combination therapy 1 year after diagnosis increased impressively from 31.0% in 2005–2009 to 77.8% in 2015–2019. This increase is especially pronounced in the patients diagnosed between 2015 and 2019, after the implementation of the new treatment guidelines supporting combination treatment as standard initiation therapy. While the use of oral PAH‐specific medication increased, the use of prostacyclin analogues intravenously or subcutaneously decreased almost by half (2005–2009: 18.4%; 2015–2019: 9.6%). Despite therapeutic developments, transplant‐free survival in the younger patients did not improve in our study. Two meta‐analyses analysed randomized controlled trials of PAH‐specific medication and described the benefit of intervention groups compared to controls (HR: 0.57, IQR: 0.35–0.93; HR: 0.56 (0.35–0.90).^27^, ^28^ However, most RCTs are placebo‐controlled or add‐on trials. There is limited evidence comparing individual PAH‐specific therapeutic agents, more specifically comparing treatment with prostacyclin analogues intravenously or subcutaneously with or without oral medication, to oral treatment regimens alone. Recent reports and guidelines recommend more aggressive treatment in the early stages of the disease to prevent progression and because of the still dismal prognosis for patients with PAH.^3^, ^4^, ^14^ It would be interesting to know whether the reduction in the use of intravenous or subcutaneous prostacyclin analogue attributed to the lack of transplant‐free survival improvement in PAH patients, especially in younger patients.

This study has several limitations. Our study endpoint was transplant‐free survival. We did not investigate the quality of life in this study. It would be interesting to know, that even though patients do not seem to live longer, whether their quality of life did improve. Given the observational design of this study, missing data are inevitable. Although with less precision, complete case analysis did not yield different outcomes in survival analysis. A clear survival benefit is only proven for epoprostenol. In our study, we did not distinguish between different prostacycline analogues, intravenous epoprostenol and intravenous or subcutaneous treprostinil. This distinction is also complicated by the fact that a noticeable amount of patients switched between these treatment modalities; switching from treprostinil subcutaneously to intravenous treatment with either treprostinil or epoprostenol, because of side effects, mainly local injection side reactions and switching from intravenous therapy to subcutaneous therapy, mainly for practical reasons. We did not have sufficient numbers of patients for each individual etiology to perform a subgroup analysis. The median follow‐up time differed between our time cohorts. This could introduce bias when patients are more at risk earlier after diagnosis. However, the proportional hazards assumption of our cox‐PH regression model holds for all included variables including the time cohort. Therefore, we consider this possible bias to be limited.

### Future perspectives

Our study did not demonstrate improved transplant‐free survival over the past 15 years of PAH treatment despite an expanded therapeutic arsenal. This leads to one of our core questions: are we treating the right patients in the right way? More detailed information is needed about PAH in the elderly population. Does this type of PAH pathophysiologically differ from PAH in younger patients? Do both groups benefit from the same medication regime? Treatment strategies in younger patients should also be evaluated. Have we become too cautious with the use of intravenous and subcutaneous prostacyclin therapy? Furthermore, we also observed that patients are still diagnosed in advanced disease stages, this continuing diagnostic delay will also attribute to a poorer prognosis. Future research is crucial for the optimization of PAH‐care to identify the right patient for the right treatment strategies.

## CONCLUSION

The 1‐, 5‐, and 10‐year transplant‐free survival rate for incident patients with PAH in our cohort was, respectively, 88%, 71%, and 35%. Treatment strategies changed over time with a significantly increased use of combination therapy. Patients older than 56 years showed a significantly worse transplant‐free survival when diagnosed between 2010 and 2019 compared to patients diagnosed between 2005 and 2009, which could be partly due to changed demographic characteristics. Patients younger than 56 years old showed neither improved nor deteriorated transplant‐free survival over time.

## AUTHOR CONTRIBUTIONS

*Conceptualization*: Paul M. Hendriks, Liza D. van de Groep, Annemien E. van den Bosch, Marco C. Post, and Karin A. Boomars. *Patient inclusion*: Robert M. Kauling, Leon M. van den Toorn, Prewesh P. Chandoesing, Annemien E. van den Bosch, Hans‐Jurgen Mager, Marco C. Post, and Karin A. Boomars. *Data curation*: Paul M. Hendriks, Diederik P. Staal, and Liza D. van de Groep. *Data analysis*: Paul M. Hendriks, Liza D. van de Groep, and Diederik P. Staal. *Supervision*: Robert M. Kauling, Leon M. van den Toorn, Prewesh P. Chandoesing, Hans‐Jurgen Mager, Annemien E. van den Bosch, Marco C. Post, and Karin A. Boomars. *Writing, review, and editing*: Paul M. Hendriks, Diederik P. Staal, Liza D. van de Groep, Robert M. Kauling, Leon M. van den Toorn, Prewesh P. Chandoesing, Annemien E. van den Bosch, Marco C. Post, and Karin A. Boomars. All authors read and approved the manuscript.

## CONFLICT OF INTEREST

The authors declare no conflict of interest.

## ETHICS STATEMENT

This study was performed according to the principles outlined in the Declaration of Helsinki. Informed consent was waived in accordance with Dutch national law. The study protocol was approved by the medical ethics committee of both participating centers (MEC‐2021‐0233, Z19.039).

## Supporting information

Supporting information.

## Data Availability

Paul M. Hendriks and Karin A. Boomars had full access to all the data in the study and take responsibility for its integrity and data analysis.
