# Supplementary material for: The evolution of survival of pulmonary arterial hypertension over 15 years
Source: Pulm Circ. 2022 Oct 1;12(4):e12137. doi: 10.1002/pul2.12137 (PMC9579738; doi:10.1002/pul2.12137)
Supplement: Supplementary file 1 — Supporting information. [file PUL2-12-e12137-s001.docx]

Supplemental material

| **Variable** | **Missing, N (%)** |
| --- | --- |
| Age at diagnosis | 0 (-) |
| Sex | 0 (-) |
| Time cohort | 0 (-) |
| Etiology | 0 (-) |
| Comorbidities | 0 (-) |
| Survival status | 0 (-) |
| NT-proBNP | 14 (4.8) |
| mRAP | 19 (6.5) |
| 6 minute walking distance | 20 (6.8) |
|  |  |

**Table S1:** Missing data per predictor

|  | 2005-2009 | 2010-2014 | 2015-2019 |
| --- | --- | --- | --- |
| Erasmus MC, Rotterdam | 26 | 50 | 69 |
| St. Antonius Hospital, Nieuwegein | 30 | 61 | 57 |

**Table S2:** Number of patients included per time cohort per participating center. P-value = 0.288.

| **Cause of death** | **Total**  **N = 151** | **2005-2009**  **N = 35** | **2010-2014**  **N = 65** | **2015-2019**  **N = 51** |
| --- | --- | --- | --- | --- |
| **Cardiopulmonary death (%)**  - RV failure  - Euthanasia (for PH)  - Sudden cardiac death  - Hemoptysis  - Pulmonary fibrosis | 95 (62.9)  - 87  - 3  - 3  - 1  - 1 | 25 (71.4)  - 22  - 2  - 1  - 0  - 0 | 38 (58.5)  - 36  - 0  - 1  - 1  - 0 | 32 (62.7)  - 29  - 1  - 1  - 0  - 0 |
| **Oncological (%)** | 10 (6.6) | 3 (8.6) | 4 (6.2) | 3 (5.9) |
| **Infectious (%)**  - Pneumonia  - Sepsis  - Other | 14 (9.3)  - 6  - 5  - 3 | 1 (2.9)  - 0  - 1  - 0 | 9 (13.8)  - 4  - 3  - 2 | 4 (7.8)  - 2  - 1  - 1 |
| **Unknown or lost to follow-up (%)** | 16 (10.6) | 2 (5.7) | 7 (10.8) | 7 (13.7) |
| **Other* (%)** | 16 (10.6) | 4 (11.4) | 7 (10.8) | 5 (9.8) |

**Table S3:** Detailed overview of causes of death per time and age cohort
*Other include: dementia, end-stage systemic sclerosis, end-stage hereditary hemorrhagic telangiectasia, gastric haemorrhage, hemorrhagic cerebrovascular accident, liver cirrhosis, multi-organ failure, renal failure, subdural hematoma, surgical complications


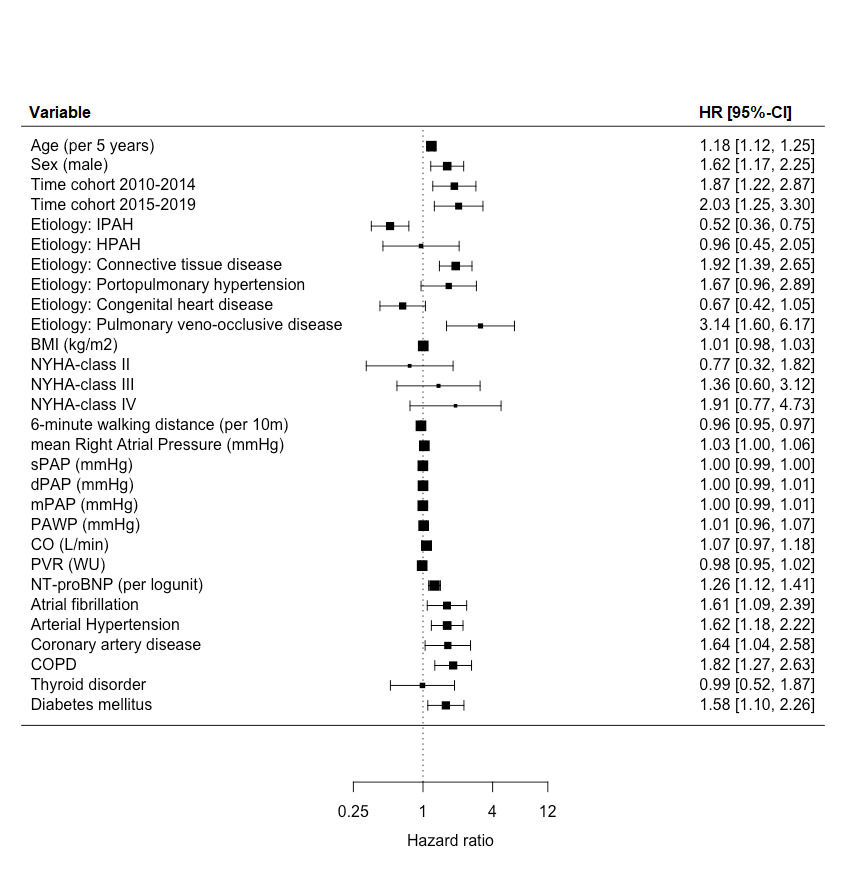


**Figure S1:** forest plot of univariable cox-PH regression models
